# Digital and Technology-Based Nutrition Interventions, Including Medically Tailored Meals (MTMs) for Older Adults in the U.S.—A Scoping Review

**DOI:** 10.3390/nu18030385

**Published:** 2026-01-24

**Authors:** Nishat Tabassum, Lesli Biediger-Friedman, Cassandra Johnson, Michelle Lane, Seanna Marceaux

**Affiliations:** 1 Nutrition and Foods Program, School of Family and Consumer Sciences, Texas State University, San Marcos, TX 78666, USA; QQC13@txstate.edu (N.T.); cassandra_johnson@txstate.edu (C.J.); ml48@txstate.edu (M.L.);; 2 Meals on Wheels Central Texas, Austin, TX 78702, USA

**Keywords:** medically tailored meals, nutrition education, digi-tech, older adults, United States

## Abstract

**Background/Objectives:** Older adults often face nutrition challenges due to mobility issues, chronic conditions, and limited access to adequate nutrition. Digital and technology-based interventions, including those with nutrition education, nutrition counseling and Medically Tailored Meals [MTMs], can help address these barriers. However, the extent and characteristics of such programs in the United States remain unclear. This scoping review aimed to map the existing evidence on digital and technology-based (“digi-tech”) nutrition interventions for older adults in the United States, with particular attention to the presence, characteristics, and gaps related to MTMs. **Methods:** This scoping review followed the PRISMA-ScR framework to map existing evidence on technology-enabled nutrition care interventions for older adults aged ≥ 60 years in the United States. Systematic searches were conducted across multiple databases, yielding 18,177 records. Following title and abstract screening, full-text review, and eligibility assessment, 16 intervention studies were included. Study designs comprised randomized controlled trials, quasi-experimental and non-randomized studies, mixed-methods feasibility studies, pilot studies, and one retrospective longitudinal cohort study. Data were extracted on study design, population characteristics, intervention components, technology modalities, outcomes, feasibility, acceptability, and reported barriers. **Results**: Interventions varied in duration [8 weeks to ≥12 months] and content. Foci ranged from remote nutrition education and mobile app-based tracking to multicomponent interventions integrating exercise, nutrition counseling, health literacy, and meal delivery. Telehealth was the most commonly used technology modality, followed by mobile health applications, wearable devices, and online educational platforms. Most interventions reported high feasibility and acceptability, with improvements in diet quality, adherence to healthy eating patterns, clinical measures such as HbA1c and blood pressure, and functional performance. Common implementation barriers included declining technology use over time, digi-tech literacy, and access to devices or the internet. Notably, no studies evaluated a digi-tech-based MTMs intervention exclusively for older adults in the U.S. **Conclusions**: Digital and technology-based nutrition interventions show promise for improving dietary and health outcomes in older adults, but there is insufficient empirical evidence. Future research might develop and evaluate hybrid digi-tech intervention models that leverage the potential of digi-tech tools while addressing barriers to technology adoption among older adults.

## 1. Introduction

The United States is undergoing a major demographic transformation, characterized by a rapidly increasing proportion of older adults [1]. Projections indicate that nearly 90 million individuals will be aged 65 years or older by 2050 [1]. This demographic shift presents profound public health challenges, particularly in managing chronic diseases, including dementia, heart disease, type 2 diabetes, arthritis, cancer, and kidney disorders [2,3]. Between 2013 and 2023, older adults in the United States continued to experience an exceptionally high burden of chronic disease. As of 2023, 93% of older adults reported having at least one chronic condition, while 80% were living with multiple chronic conditions [4]. Adequate nutrition is essential for preventing disease progression, maintaining functional ability, and promoting independence [2]. However, many older adults experience difficulties in accessing or preparing healthy meals, which can result in inadequate dietary intake, nutrient deficiencies, and poor management of chronic diseases such as diabetes and hypertension [5].

To address these challenges, the Older Americans Act [OAA], originally passed in 1965, established a framework for community-based services that support aging in place [6]. One of the most vital provisions of OAA is the Senior Nutrition Program [SNP], which includes congregate meal services [CMSs] and home-delivered meals [HDMs] for older adults. The national meal programs that are funded through the OAA have long played a vital role in supplying nutritious meals to older adults [7,8,9,10,11]. However, existing meal programs may lack personalization, medical tailoring, and digital and technological integration [12]. A nationwide survey of participants in Older Americans Act [OAA] programs revealed that 57% of individuals enrolled in the Congregate Meal Program [CMP] are living with five or more chronic health conditions [13,14]. Additionally, nearly one-third [32%] of CMP participants report taking six or more prescription medications daily to address these various health issues [11]. A systematic review of 80 studies highlighted the need for more rigorous research to refine home-delivered meal models and expand these services to all eligible older adults [10,12].

Nutrition interventions such as nutrition education and nutrition counseling play a pivotal role in promoting healthy aging and reducing the risk and impact of chronic diseases [15]. Evidence suggests that older adults who receive structured nutrition education are more likely to improve diet quality, maintain muscle mass, and manage conditions such as diabetes, cardiovascular disease, and obesity [16,17]. Moreover, incorporating technology-based approaches such as telemonitoring, mobile applications, and virtual counseling has been shown to increase accessibility, engagement, and adherence among older populations, including those in rural or under-served areas [16,18]. Digital health interventions, including nutrition education delivered via telehealth or apps have shown promise in supporting dietary behaviors and chronic disease management. For example, Batsis et al. (2021) implemented a multi-component, technology-based rural weight management program that combined weekly nutrition counseling with remote monitoring, finding it both feasible and well-accepted among older adults with obesity, with notable improvements in diet quality, weight, and physical function [16].

MTMs are a recognized “Food is Medicine” strategy, designed by registered dietitian nutritionists to meet the medical and nutritional needs of individuals with chronic illnesses such as diabetes, heart disease, and kidney disorders [19,20]. These meals are specifically formulated to provide optimal nutrient intake while addressing dietary restrictions, medication interactions, and disease-specific nutritional guidelines [21]. Evidence indicates that participation in MTM programs is associated with reduced hospital admissions, lower healthcare costs, and improved health outcomes [19,22,23,24,25]. A study reported that older adults participating in a home-delivered MTMs program experienced reduced malnutrition risk and improvements in blood pressure, hemoglobin A1C, and BMI over time [26]. Another study estimated that providing MTMs to eligible older adults could prevent over a million hospitalizations annually and save more than USD 13 billion in healthcare costs nationwide [20]. Unlike traditional home-delivered meals and congregate meal programs, which primarily aim to support general nutrition and food security [7,8], MTMs are tailored to individual diagnoses, comorbidities, and treatment plans and are integrated into broader models of clinical care [19,20]. In this scoping review, MTMs are analytically distinguished from traditional home-delivered meals and congregate meal programs. This distinction is necessary because many nutrition service models for older adults provide meals that support general dietary adequacy and food security but are not designed to meet individualized medical or therapeutic dietary needs. Clarifying this boundary allows for a more accurate interpretation of the evidence and helps identify gaps in the evaluation of technology-based MTMs specifically designed for older adults in the United States.

Digital and technological tools, described as digi-tech-based approaches, may be used to support a range of functions in nutrition interventions, including the delivery of nutrition services, participant monitoring and tracking, recruitment and engagement, communication between participants and providers, or logistical coordination such as meal ordering and scheduling [16,22,27]. In this review, the term digi-tech based refers to digital and technological tools actively supporting or facilitating program delivery rather than serving solely as data collection or evaluation tools [16,22,28]. These technological functions are not mutually exclusive and may occur in combination within a single intervention. Because each function has different implications for feasibility, scalability, and implementation, the role of technology within each included study was documented and considered in the synthesis [16,22,27,28].

Integrating digital platforms, nutrition interventions including nutrition education, nutrition counseling and MTMs for older adults may streamline meal ordering and tailored based on individual health needs [16,22,27,28]. This combined approach not only ensures consistent access to medically appropriate meals but also can provide ongoing dietary education to improve behavior change, support disease self-management, and promote long-term adherence [27,28,29,30]. Furthermore, technology integration can enhance communication between clients, dietitians, and healthcare providers, enable real-time monitoring of dietary intake and health indicators, and extend program reach to underserved or rural areas where in-person services are limited [16,27]. Despite the promise of these strategies, it remains unclear how extensively digi-tech-based nutrition interventions, including nutrition education and MTMs, have been implemented for older adults in the United States. This scoping review does not seek to compare MTMs with nutrition education interventions. Instead, it maps digi-tech-based approaches to nutrition interventions for older adults in the United States, with particular attention to the presence characteristics of MTM integration. The goal is to explore the opportunities, challenges, and directions for innovation in policies and programs for older adults.

## 2. Materials and Methods

### 2.1. Study Design

This scoping review was conducted to systematically explore and summarize the evidence on technology-delivered medically tailored meals [MTMs] and nutrition education for older adults in the United States. This scoping review was conducted using the framework outlined by Arksey and O’Malley (2005) [31], incorporating additional guidance developed by Levac and colleagues [32]. The process included five key stages: (1) identifying the research question; (2) identifying relevant studies; (3) selecting studies based on pre-defined eligibility criteria; (4) charting the data; and (5) collating, summarizing, and reporting the results. An iterative approach was used, allowing for refinement of the search strategy and data extraction form as the review progressed. Reporting follows the Preferred Reporting Items for Systematic Reviews and Meta-Analyses extension for Scoping Reviews [PRISMA-ScR] guidelines [33] to ensure transparency and reproducibility.

### 2.2. Identifying the Research Question

This scoping review was guided by the following research question: “What evidence exists on technology-based nutrition intervention, and to what extent have medically tailored meals been implemented or evaluated within these technology-supported models, including the types of technologies used, their feasibility, and the challenges as-sociated with implementation, and what gaps remain for future research and program development?” This question was formulated using the Population–Concept–Context [PCC] framework recommended for scoping reviews [33]. The population of this scoping review are adults aged 60 years and older residing in the United States. The concept focuses on nutrition interventions delivered through technology, specifically medically tailored meal programs and nutrition education delivered via mobile applications, telehealth platforms, or other digital tools. The context includes interventions implemented in community-based, healthcare-affiliated, or home-delivered meal program settings across the United States.

To guide the review process, the research question was further refined into the following sub-questions:

1. What types of digital or technological [digi-tech] approaches are used in nutrition interventions, including nutrition education and MTMs, for older adults in the U.S.?
2. What evidence exists regarding the feasibility, acceptability, and effectiveness of these interventions?
3. What challenges and barriers have been reported in implementing digi-tech-based nutrition interventions for older adults in the U.S.?
4. What are the gaps or opportunities to inform future intervention development for older adults in the U.S.?

### 2.3. Eligibility Criteria

For this review, we included primary research studies that implemented on nutrition interventions with older adults, defined as individuals aged 60 years or above [34] and living in the United States. Initially, the review was restricted to studies that exclusively targeted older adults [≥60 years] in the United States participating in digi-tech-based MTM interventions. However, preliminary searches identified only a limited number of eligible studies. To better reflect current intervention models, the inclusion criteria were expanded to incorporate a range of nutrition interventions beyond MTMs and interventions involving mixed adult populations, where older adults represented a significant subgroup and studies reported results for older adults as a subgroup. A significant subgroup of older adults was defined as studies in which eligibility criteria restricted enrollment to adults aged 60 years or older, where the mean or median participant age was at least 60 years, or where outcomes were reported separately for older adults through stratified or subgroup analyses. This definition reflects the age distributions and reporting practices of the included studies, many of which explicitly targeted older adults through age-based eligibility criteria or enrolled predominantly older samples, while allowing for the inclusion of nutrition interventions delivered through digital or technology platforms including but not limited to mobile applications, telehealth services, web-based portals, or wearable devices. Interventions could involve MTMs and/or technology-based nutrition education or counseling; however, meal programs were required to be medically tailored when a meal component was included. Eligible interventions involved nutrition intervention delivered through a technology-based platform, such as mobile applications, telehealth services, web-based portals, wearable devices, or other digital tools. Studies were required to report at least one relevant nutrition outcome, which could include measures of feasibility, usability, accessibility, dietary intake, health outcomes, adherence, cost-effectiveness, or user experience. Secondary and tertiary research studies related to this topic, including reviews and protocols, were excluded from the studies to be summarized. However, the authors retained relevant papers for interpreting the results of this scoping review. Only studies published in English between January 2015 and December 2024 were considered. Studies were excluded if they were secondary or tertiary research [i.e., review articles], abstracts without a full text version, protocols without results or results for a significant older adult subgroup, or other types of articles like, commentaries or editorials.

### 2.4. Search Strategy

A comprehensive search strategy was developed to identify relevant literature. The databases searched were PubMed, MEDLINE, Google Scholar CINAHL, Web of Science, Scopus, and Cochrane CENTRAL. Searches incorporated both controlled vocabulary [MeSH terms] and free-text keywords to capture the core concepts of digital and technology-based interventions, medically tailored meals and nutrition education, older adult populations, and the U.S. context. The terms used included, for example, telemedicine, mobile applications, technology, telehealth, mobile health, mHealth, eHealth, digital intervention, internet-based, web-based, app, apps, smartphone, nutrition therapy, nutrition education, diet therapy, tailored nutrition, medically tailored meals, tailored meal, individualized nutrition, senior nutrition program, diet counseling, dietary education, older adults, seniors, aged, and United States; U.S. Boolean operators [“AND”/“OR”], truncation, and phrase searching were applied to combine terms, and search strings were adapted for each database. Filters were applied to limit the results to English-language publications from January 2015 to December 2024 and to studies conducted in the United States. The search was limited to studies published in English between January 2015 and December 2024 to capture the most recent evidence reflecting current technology use and was filtered to include only studies conducted in the United States. Details of the full search strategy are provided in Appendix A, Table A1: Complete search strategy for the databases.

### 2.5. Study Selection

The search results from the selected databases were first imported into EndNote [version 20.6] to identify and remove duplicate records. The deduplicated list was then uploaded into Covidence for the screening process. Screening was completed in two stages. In the first stage, titles and abstracts were reviewed to remove studies that clearly did not meet the inclusion criteria. In the second stage, the full texts of potentially eligible studies were examined in detail to confirm their suitability for inclusion. Two reviewers conducted each stage independently, and any disagreements were addressed through discussion. Google Scholar returned 18,100 records; however, due to the extremely high volume of duplicates and non-scholarly entries typically retrieved from this database, only the first 1000 results ranked by relevance were screened. This approach was used in prior scoping reviews when managing exceptionally large result sets retrieved from Google Scholar and is intended to capture the most relevant and frequently cited literature [35,36]. However, this decision may have resulted in the exclusion of some eligible studies appearing beyond the initial results and therefore introduces potential selection bias. This limitation is acknowledged and considered when interpreting the findings of the review. Despite this limitation, the use of multiple databases, predefined eligibility criteria, and independent screening procedures strengthens the confidence that the review captured the most relevant and influential studies addressing technology-based nutrition interventions for older adults. Regular collaborator meetings were held during the review process to resolve conflicts, with team members presenting their rationale for including or excluding specific studies. If consensus could not be reached during these discussions, a third reviewer provided the final decision. Reasons for exclusion at the full-text stage were recorded systematically. The study selection process followed an iterative approach, allowing for minor adjustments to the eligibility criteria as familiarity with the literature increased. The process is summarized in a PRISMA-ScR flow diagram, which details the number of records identified, screened, excluded, and included in the final synthesis.

### 2.6. Data Extraction

The first author extracted data from all included studies into a Microsoft Excel spreadsheet. The following information was recorded for each study: citation details [first author, year], study design, data collection method, and details of the intervention. Intervention-related fields included the type of MTMs program when applicable, nutrition education component, and the type of digital or technology-based approach [e.g., telehealth, mobile application, web-based platform, wearable devices]. Outcome-related data included the primary and secondary outcomes measured [e.g., feasibility, usability, accessibility, dietary intake, clinical health indicators, adherence, cost-effectiveness, user satisfaction] and key findings or results. Where available, the author extracted information on implementation factors such as reported barriers, facilitators, and recommendations for future practice. The key characteristics extracted from the included studies, including study design, intervention components, technology type, and outcomes, are summarized in Appendix B, Table A2: Summary of included studies on digital or technology-based nutrition interventions for older adults in the U.S.

## 3. Results

### 3.1. Study Characteristics

**Figure 1:**
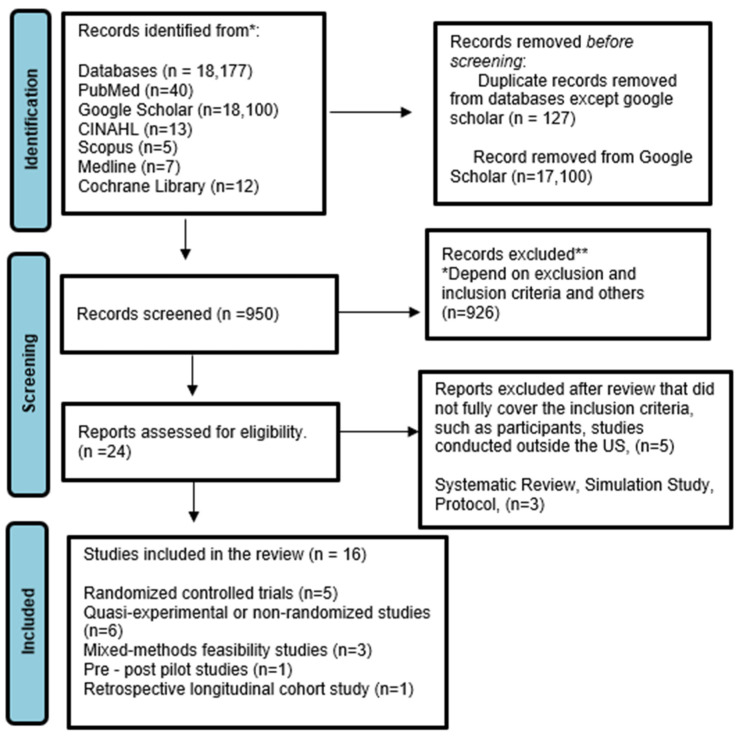
PRISMA flow diagram of study selection. *An asterisk* (*) indicates records identified from database searches prior to duplicate removal and screening. *A double asterisk* (**) indicates records excluded during title/abstract and full-text screening based on predefined inclusion and exclusion criteria. These notations are part of the standard PRISMA-ScR flow diagram template and are included to enhance transparency in the study selection process.

This scoping review included examining 16 digital or technology-based nutrition interventions [16,22,29,37,38,39,40,41,42,43,44,45,46,47,48,49] like nutrition education, nutrition counseling, and lifestyle changes-related interventions for older adults in the U.S. The review initially sought to identify digi-tech-based nutrition interventions with MTM integration; however, no included study implemented this kind of intervention exclusively for older adults. Instead, the included studies primarily focused on digi-tech-based interventions focused on nutrition education, counseling, self-monitoring, or broader individualized nutrition solutions. The 16 included intervention studies employed a range of methodological designs. These included randomized controlled trials [*n* = 5] [22,37,38,39,40], quasi-experimental or non-randomized intervention studies [*n* = 6] [16,29,41,42,43,44], mixed-methods feasibility studies [*n* = 3] [45,46,47], one pre–post pilot study [*n* = 1] [48], and one retrospective longitudinal cohort study [*n* = 1] [49]. Each study was assigned a single primary design classification, which was applied consistently across the Results text, summary tables, and figures to ensure internal consistency and transparency. Study designs were classified using standardized methodological definitions. Randomized controlled trials were defined as studies with random allocation to intervention conditions; quasi-experimental studies included non-randomized or single-arm intervention designs; mixed-methods feasibility studies combined quantitative and qualitative assessments of feasibility or acceptability; pilot studies were small-scale preliminary intervention evaluations; cohort studies involved observational longitudinal follow-up. Each article included was assigned a single primary design classification, which was applied consistently across the Results text, tables, and figures. Sample sizes varied substantially across the included studies, ranging from small feasibility and pilot studies enrolling approximately 15–30 participants to large intervention evaluations involving up to 1977 participants. Interventions targeted older adults with diverse health profiles, including those with chronic conditions such as diabetes, hypertension, cardiovascular disease, and HIV, as well as individuals experiencing food insecurity or frailty [16,22,29,37,38,39,40,41,42,43,44,45,46,47,48,49]. The complete study selection process, including the records identified, screened, excluded, and included in the review, is illustrated in Figure 1 [PRISMA flow diagram].

### 3.2. Intervention Characteristics

No included study evaluated a digi-tech-based medically tailored meals [MTMs] intervention designed exclusively for older adults in the United States. The 16 included studies [16,22,29,37,38,39,40,41,42,43,44,45,46,47,48,49] varied widely in duration, delivery format, and content. Based on program length, they can be grouped into three categories:

Short-term interventions [≤16 weeks] often delivered intensive nutrition-related support, including dietary counseling, cooking skills training, tailored education, or behavior change strategies [39,44,45]. For instance, a study included an 8-week remote dietary counseling program for older adults with hypertension, which combined weekly registered dietitian nutritionist [RDN] calls with mobile app dietary monitoring, an educational website, daily health tips, and goal-reinforcement messages, resulting in improved vegetable, legume, and nut intake and reduced processed food consumption [45]; a 16-week web-based cooking demonstration series emphasizing protein intake, targeting muscle mass preservation, protein consumption, and cooking confidence [39]; the mHealth-based diabetes self-management program by Dugas et al. ran for 13 weeks with extended follow-up and used Samsung tablets, Fitbit devices, and a custom diabetes app to improve HbA1C and self-management behaviors [41]; and an 8-week intensive tele-counseling program paired weekly RD calls with app-based dietary tracking via MyFitnessPal, daily nutrition tips, tailored feedback emails, and an educational website; participants also received maintenance counseling at 5 and 11 months, leading to reductions in sodium in-take, improved Healthy Eating Index scores, and lowered blood pressure [44]. Other short-term interventions included a 10-week post-discharge MTMs program with optional telehealth nutrition counseling to reduce hospitalization and mortality [22], the “Together in Care” program providing daily MTM delivery, home safety inspections, medical supply provision, social engagement, and symptom monitoring via tablet devices for 3 months post-discharge [48]. The Olitorile app pilot study delivered feedback over 12 weeks, provided personalized recipes, dietary tracking, in-app messaging, and self-monitoring features, resulting in significant improvements in Mediterranean diet adherence and legume intake among older adults with frailty [40]. Similarly, another study evaluated a telephone-based nutrition education and counseling program [47].

Medium-term interventions [4–6 months] often integrated nutrition support with physical activity or chronic disease management, monitoring changes in diet, health status, and functional performance. Batsis et al. conducted a 26-week weight management program that combined weekly individual video-based dietitian sessions, on-site group meetings, and twice-weekly virtual exercise classes, supplemented by aerobic activity prescriptions and Fitbit-based self-monitoring. This program targeted older adults with obesity and monitored changes in body weight, physical function, and dietary quality [16]. A 6-month multicomponent lifestyle program incorporated regular virtual dietitian sessions, in-person group nutrition classes, and structured physical activity led by physical therapists, supported by remote monitoring via wearable devices [43]. This intervention emphasized both weight loss and the preservation of muscle mass, with outcomes measured through body composition analysis, diet quality assessment, and functional performance tests [43]. Additionally, Rodriguez et al. provided a 6-month telephone-based counseling program for veterans with hypertension provided monthly stage-of-change-tailored nutrition counseling calls, focusing on adherence to the DASH diet [38]. Lee et al. tested a 6-month flexible congregate meal program with three delivery models: virtual [grab-and-go meals + online activities], hybrid [virtual + in-person], and traditional in-person congregate meals [42].

Longer-term interventions [≥12 months] can be categorized as real-world, large-scale, or observational programs with extended follow-up, focusing on sustained health and nutrition outcomes. For example, Yu et al. offered a year-long Food Access Pilot Project delivering medically supportive meals, pantry boxes, and RD phone support [49]. Other longer-term studies included the digital health feasibility study by LoBuono et al. (2021) assessing nutrition-related care in older adults with Parkinson’s disease [46].

Notably, no included studies directly evaluated a digital technology-based MTMs program specifically designed for older adults in the U.S. While some interventions integrated meal delivery with technology, such as “Together in Care” [48] and the “Food Access Pilot Project” [49], these were not exclusively targeted to older adults, nor did they assess MTM delivery as the primary technology-based intervention.

### 3.3. Technology Modalities

**Figure 2:**
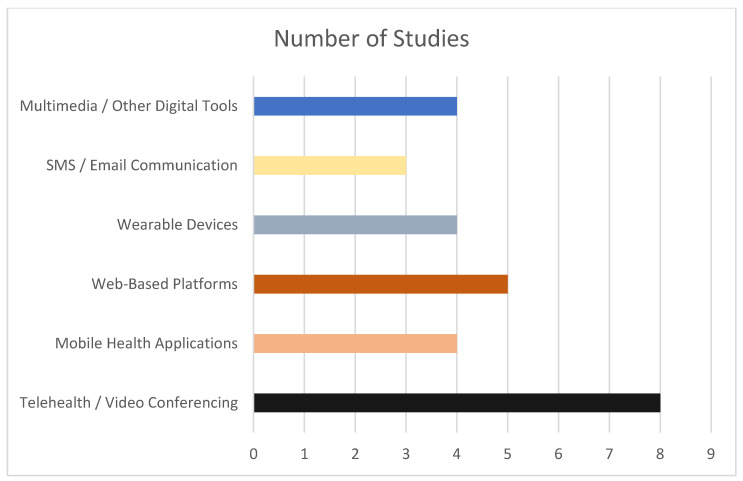
The frequency of technology modalities used across interventions in the included studies. The frequency of technology modalities used across the included studies (*n* = 16). Telehealth and video conferencing were the most used modalities, followed by web-based platforms, mobile health applications, wearable devices, SMS/email communication, and other digital tools. Technology categories were not mutually exclusive, and several studies incorporated more than one modality.

According to the studies included, technology played a central role in delivering, monitoring, and supporting interventions. Telehealth and video conferencing were the most common modalities used in included studies [16,22,29,38,40,42,47]. Telehealth platforms range from general-purpose video conferencing to specialized secure systems. For example, Batsis et al. used a HIPAA-compliant Zoom platform for weekly one-on-one RDN counseling and twice-weekly group exercise classes, ensuring both security and ease of access for older adult participants [16]. These platforms enable real-time interaction between participants and RDN health educators or exercise specialists, and reduce barriers related to travel, mobility, and geographic location. In several studies, telehealth was used to deliver structured exercise classes in addition to dietary counseling [16,43]. Mobile health applications were integrated into at least six interventions and were often paired with other delivery methods. These apps supported dietary self-monitoring, provided tailored feedback, reinforced health goals, and in some cases, delivered educational messages [40,41,43,44,45]. For instance, Chang et al. used both a study-specific and a commercial dietary monitoring app [45], while Dugas et al. deployed a custom mobile health diabetes self-management platform [Diasocial] integrated with activity tracking [41]. In addition, Schrauben et al. used the MyFitnessPal app to enable detailed dietary tracking linked to RDN counseling [44], and Su et al. employed the Olitor app to enhance adherence to the Mediterranean diet with personalized recipes, in-app messaging, and progress tracking [40]. Wearable devices were also incorporated in six studies to capture objective measures of physical activity, step count, and sometimes sleep patterns or heart rate [16,39,41,43,46]. These devices provided both participants and intervention staff with immediate feedback and allowed for remote performance monitoring. In interventions like Batsis et al., wearable data were aggregated via platforms such as Fitabase for clinician review and program tailoring [16]. Online portals and educational websites were also used in several included interventions [40,42,44,45]. Moreover, electronic tablets or smartphones were provided in four studies [16,41,46,48]. Less commonly, studies explored innovative formats such as a DVD-based interactive eHealth program that combined tailored feedback with survivor narratives to motivate dietary change [37]. The distribution of technology types across studies is illustrated in Figure 2:

### 3.4. Reported Outcomes and Key Findings

Feasibility was reported in most included studies with strong recruitment and retention in many cases. Structured programs such as Batsis et al. [16], Wood et al. [43], and Chang et al. [45] achieved completion rates exceeding 80%, while longer interventions like Schrauben et al. [44] reported higher attrition, with 43% dropout at 12 months. Technology adherence also depends on the type of tool. Wearable devices, for example, were used consistently on 70–85% of days in Batsis et al. [16] and Salas-Groves et al. [39], while mobile app use tended to drop over time, as seen in Chang et al. [45] and Shah et al. [29]. Participants in several studies also encountered challenges with device setup, app navigation, and internet connectivity, particularly those with limited digital skills [46,48]. Most interventions were well-received [16,40,44,45]. In Chang et al. [45], over 90% of participants rated RDN counseling as highly satisfactory and Batsis et al. [16] reported a 4.7/5 satisfaction score.

Dietary improvements and overall diet quality were common in several studies [39,40,43,44,45]. Adherence to the Mediterranean and DASH diets improved, alongside reductions in processed food and sodium intake [20,29,40,45]. Clinical benefits included lower HbA1C in older adults with type 2 diabetes [41], improved blood pressure, and Healthy Eating Index scores [44]. Some studies noted metabolic improvements, such as insulin resistance, lipid profiles, and inflammation, though not all were significant [39,40]. Healthcare utilization results were mixed; the Together in Care program reduced costs, readmissions, and ICU stays, especially for COPD patients [48], while a large RCT of post-discharge MTMs showed no difference in all-cause hospitalization but reduced mortality and heart failure admissions in subgroups [22].

## 4. Discussion

This scoping review mapped the existing evidence on technology-integrated nutrition education for older adults in the U.S. and highlights the range of intervention types, technology modalities, feasibility, acceptability, and reported outcomes. While the 16 studies included demonstrate the breadth of technology-delivered nutrition strategies for older adults, a key finding is the lack of interventions specifically delivering MTMs exclusively through technology to this population. The interventions identified in this review varied widely in duration, scope, and delivery [16,37,43,45]. Telehealth was the most frequently used modality, supporting both individualized dietitian counseling and, in some cases, group-based nutrition or exercise sessions [16,20,40,45]. Mobile health applications, wearable activity trackers, online portals, and smartphones/tablets were also used to enable self-monitoring, deliver educational content, and facilitate remote engagement [39,41,44,45]. This diversity of approaches demonstrates the adaptability of digital health tools in addressing nutrition needs among older adults but also highlights the fragmented nature of the evidence. Interventions largely prioritized nutrition education, counseling, and lifestyle behavior change, with limited exploration of digitally enabled MTM models that could combine the clinical benefits of tailored meal provision with the scalability of telehealth and remote monitoring.

Across studies, interventions primarily emphasized nutrition education, counseling, and lifestyle behavior change, with no exploration of technology-delivered MTM models that integrate individualized meal provision with technology-based delivery or monitoring [39,41,44,45]. This absence likely reflects the relative novelty and complexity of integrating clinically tailored meal provision with digital health infrastructure for older populations, rather than a lack of interest in MTMs or technology-delivered nutrition care. The lack of evaluated digital technology-based MTM programs specifically targeting older adults in the U.S. appears to stem from multiple intersecting challenges related to policy, operations, clinical integration, and technology [50,51]. Reimbursement for MTMs is not consistently established across payers and often varies by benefit structure, which can constrain program sustainability and limit the investment in digital systems needed to support technology-enabled delivery. Clinical integration poses additional challenges, as effective MTM delivery typically requires coordination among healthcare providers, registered dietitian nutritionists, and meal service organizations, along with timely access to relevant clinical information [52]. Establishing these connections can be difficult within fragmented healthcare systems that lack standardized pathways for nutrition referral and care coordination. Technology-related barriers further complicated implementation for older adults, including limited digital literacy, inconsistent access to devices, and unreliable broadband connectivity [53]. These challenges are often more pronounced in rural and economically disadvantaged communities [53]. Taken together, these factors help explain why technology-based MTMs have not yet been rigorously evaluated for older adults and highlight the need for implementation-focused research that emphasizes feasible care integration, aligned reimbursement structures, and equity-centered technology design.

Most interventions demonstrated feasibility, with strong recruitment and retention rates in short- and medium-term programs [16,43,45]. High satisfaction ratings were noted, such as over 90% of participants rating dietitian sessions as “very” or “extremely” satisfactory in a study [16]. These findings suggest that technology-based nutrition interventions are well-accepted by older adults when they are personalized and interactive. However, consistent barriers emerged: for example, lower digital literacy among older adults [48]. Mobile app engagement tended to decline over time, particularly in interventions without regular synchronous contact [29,45]. These barriers highlight the need for technical support and digital skills training as integral components of program design for older adults. Addressing these barriers is critical for equity, as they may disproportionately affect rural, low-income, or marginalized older adults.

Barriers related to digital literacy, access to devices, and declining engagement may disproportionately affect individuals living in rural areas, those with lower incomes, and older adults with limited access to reliable broadband internet [46,48]. Rural residence may further intensify these challenges due to infrastructure limitations and fewer opportunities for in-person technical assistance, while financial constraints may restrict access to smartphones, tablets, or data plans required for participation [46,48]. Without intentional design strategies to address these structural barriers, technology-based nutrition programs risk widening existing disparities in access to nutrition care and chronic disease support. Strategies such as low-bandwidth intervention options, device provision, individualized training, and flexible modes of engagement have been identified as important approaches for promoting more equitable access and sustained participation among older adults [47,54]. 

Taken together, these findings highlight several practical design principles for technology-based nutrition interventions targeting older adults [16,22,29,37,38,39,40,41,42,43,44,45,46,47,48,49] Regular synchronous contact, such as scheduled telephone- or video-based counseling appears to support sustained engagement and participant satisfaction. Structured onboarding processes, ongoing technical support, and clear guidance on how to use devices or applications are also essential for minimizing barriers related to technology adoption [16,37]. In several studies, providing participants with devices, including tablets or wearable activity trackers, facilitated participation among older adults who might otherwise lack access to appropriate technology [16,41,43]. These observations suggest that hybrid intervention models, which combine digital tools with consistent human support, may be particularly well-suited to the needs and preferences of older adult populations.

The evidence suggests that technology-delivered nutrition interventions can positively influence dietary behaviors, clinical outcomes, hospital costs, and readmissions [16,41,45,48]. These findings suggest that the impact of technology-enabled nutrition education on healthcare utilization may depend on population characteristics, intervention intensity, and integration with broader care systems. 

## 5. Strengths

This scoping review has several important strengths. To begin with, it focuses on a timely and relevant topic by exploring how technology is being used to deliver and improve nutrition interventions for adults aged 60 years and older in the United States [16,22,39,45,48]. This is especially important as technology becomes a larger part of healthcare, and Food is Medicine programs continue to grow and aim to better support older adults with chronic health conditions. Another strength of this scoping review is its rigorous and transparent approach. This scoping review followed well-recognized frameworks, including the Arksey and O’Malley model, Levac et al.’s refinements, and the PRISMA-ScR checklist [31,32]. These methods provided a clear structure for identifying, selecting, and analyzing the literature, helping ensure that the review process was systematic. In addition, the review included studies from multiple database sources published between 2015 and 2024. This wide search captured a broad and current picture of how technology is being used in nutrition interventions for older adults. The review also provides a clear and organized summary of different technology types, including telehealth, mobile applications, wearable activity trackers, and online platforms [16,22,29,37,38,39,40,41,42,43,44,45,46,47,48,49]. It highlights how these tools have been used to support dietary behavior change, monitor health outcomes, and improve access to nutrition care for older populations. Finally, the review identifies key gaps in the evidence, such as the lack of studies that directly evaluate fully-technology-delivered MTM programs for older adults. It also offers useful guidance for future research and program development, emphasizing the need for hybrid models that combine digital tools with personalized nutrition care. Together, these strengths make this review a valuable contribution to understanding how technology can support nutrition interventions for older adults and guide future research and policy in this area.

## 6. Limitations

This scoping review has several limitations that should be considered when interpreting the findings. First, while the search strategy was comprehensive, it was limited to studies conducted in the United States and published in English, which may have excluded relevant evidence from other contexts or non-English sources. Second, the included studies varied widely in their design, intervention components, technology modalities, duration, and outcome measures that can make direct comparisons challenging [16,39]. Third, the quality and rigor of evidence were inconsistent, while some randomized controlled trials provided robust findings [22,38], many studies were small-scale feasibility or pilot trials with limited generalizability [40,47].

Another limitation of this scoping review is the decision to screen only the first 1000 records retrieved from Google Scholar. This decision may have led to the exclusion of some eligible studies appearing beyond the initial search results and may introduce selection bias. Moreover, combined technology-based components with in-person elements made it difficult to separate the impact of the technology itself [43]. Furthermore, only a few studies directly addressed MTM delivery, and none rigorously evaluated a technology-delivered MTMs program exclusively for older adults in the US [50,51], highlighting a major evidence gap. Finally, most studies relied on self-reported measures for dietary intake, physical activity, and satisfaction, which may be subject to recall and social desirability bias [46,53].

## 7. Conclusions

This scoping review found that while a variety of technology modalities, including telehealth, mobile applications, wearable devices, and online portals are being used to de-liver nutrition education and lifestyle interventions for older adults in the United States [16,22,46,50], no studies directly evaluated a digital technology-delivered MTMs program specifically designed for this population. Existing evidence supports the feasibility, acceptability, and potential health benefits of technology-enabled nutrition interventions, but most focus on education and counseling rather than MTM delivery [24,46].

The findings have direct implications for existing and emerging nutrition programs and policies. For community-based organizations such as Meals on Wheels, integrating technology to support screening, referral, monitoring, and coordination could enhance program reach and operational efficiency while preserving the human-centered delivery model that is central to serving older adults. Within Medicare Advantage, where supplemental benefits increasingly include nutrition services, the absence of evaluated digital technology-based MTMs for older adults underscores the need for pilot programs and rigorous evaluation to inform benefit design, scalability, and cost-effectiveness. More broadly, for Food is Medicine initiatives, this review underscores the opportunity to leverage technology not only for nutrition education or other nutrition interventions, but also to support the integration of medically appropriate meal provision within broader care coordination and monitoring frameworks for older adults. In practice, digital technology-based MTMs could include digital platforms that allow clients or caregivers to select medically appropriate meals based on diagnosed conditions, communicate dietary preferences or restrictions, receive tailored nutrition guidance, and provide ongoing feedback on meal satisfaction and health-related outcomes through websites, mobile applications, or other accessible technologies.

## Data Availability

No new data were created or analyzed in this study.

## Abbreviations

The following abbreviations are used in this manuscript:

BMI	Body Mass Index
CMSs	Congregate Meal Services
CMP	Congregate Meal Program
COPD	Chronic Obstructive Pulmonary Disease
DASH	Dietary Approaches to Stop Hypertension
HbA1C	Hemoglobin A1c
HDMs	Home-Delivered Meals
HEI	Healthy Eating Index
HIPAA	Health Insurance Portability and Accountability Act
ICU	Intensive Care Unit
mHealth	Mobile Health
MTMs	Medically Tailored Meals
OAA	Older Americans Act
PCC	Population–Concept–Context
PRISMA-ScR	Preferred Reporting Items for Systematic Reviews and Meta-Analyses Extension for Scoping Reviews
RCT	Randomized Controlled Trial
RD	Registered Dietitian
RDN	Registered Dietitian Nutritionist
RDNs	Registered Dietitian Nutritionists
RCTs	Randomized Controlled Trials
U.S.	United States
Digi-tech	Digital Technology
WHO	World Health Organization

## Appendix A

**Table A1:** The complete search strategy for the databases.

Database	Search Strategy
**PubMed**	(“Telemedicine” [MeSH] OR “Mobile Applications” [MeSH] OR “technology” [tiab] OR “telehealth” [tiab] OR “telemedicine” [tiab] OR “mobile health” [tiab] OR “mHealth” [tiab] OR “eHealth” [tiab] OR “digital intervention” [tiab] OR “internet-based” [tiab] OR “web-based” [tiab] OR “app” [tiab] OR “apps” [tiab] OR “smartphone” [tiab])AND(“Nutrition Therapy” [MeSH] OR “Nutrition Education” [MeSH] OR “Diet Therapy” [MeSH] OR “tailored nutrition” [tiab] OR “medically tailored meals” [tiab] OR “tailored meal” [tiab] OR “individualized nutrition” [tiab] OR “senior nutrition program” [tiab] OR “nutrition education” [tiab] OR “diet counseling” [tiab] OR “dietary education” [tiab])AND(“Aged” [MeSH] OR “Older Adults” [tiab] OR “senior” [tiab] OR “seniors” [tiab] OR “elderly” [tiab]) AND (“United States” [MeSH] OR “United States” [tiab] OR “USA” [tiab])
**Scopus**	TITLE-ABS-KEY (elderly OR “older adults” OR seniors OR geriatric) AND TITLE-ABS-KEY (“nutrition education” OR “dietary counseling” OR “dietary education” OR “diet therapy”) AND TITLE-ABS-KEY (technology OR telehealth OR “mobile health” OR mHealth OR eHealth OR “digital intervention” OR “internet-based” OR “web-based” OR app OR apps OR smartphone) AND TITLE-ABS-KEY (tailored OR personalized OR individualized OR customized)
**Google Scholar**	(United States* OR U.S. OR US OR USA) AND (Older adults* OR senior OR aging population) AND (technology OR mobile app OR technology delivered) AND (senior nutrition program OR medically tailored meal OR nutrition OR nutrition education OR nutrition intervention)
**Web of Science**	((“older adults” OR elderly OR seniors OR geriatric) AND (“technology” OR “mobile health” OR mHealth OR eHealth OR “digital intervention” OR online OR “internet-based” OR “web-based” OR app OR apps OR smartphone OR virtual) AND (“tailored nutrition” OR “medically tailored meals” OR “nutrition education” OR “diet counseling” OR “dietary education” OR “nutrition intervention” OR “nutrition program” OR “diet therapy” OR “nutrition therapy”) AND (“United States” OR USA OR America))
**Medline**	(“older adults” OR elderly OR “older people” OR seniors OR geriatric) AND (“medically tailored meals” OR “nutrition intervention” OR “nutrition education” OR “Tailored Nutrition”) AND (technology OR telehealth OR telemedicine OR digital OR virtual OR app OR online OR eHealth OR mHealth) AND (United States OR USA OR America)
**Cochrane Library**	(“elderly” OR “older adults” OR “seniors” OR geriatric) AND (“nutrition education” OR “dietary counseling” OR “diet therapy”) AND (“technologically Nutrition” OR “Mobile app nutrition” OR “mobile health” OR mHealth OR eHealth OR “digital nutrition intervention” OR “internet-based nutrition” OR “web-based” OR “app” OR apps OR “smartphone Nutrition” OR “technologically tailored Nutrition” personalized OR “technologically individualized Nutrition” OR “customized Diet”)
**CINAHL**	(MH “Aged+”) OR “elderly” OR “older adults” OR “seniors” OR geriatric) AND (“technology” OR telehealth OR “mobile health” OR mHealth OR eHealth OR “digital intervention” OR “internet-based Nutrition” OR “web-based nutrition” OR app OR apps OR “smartphone nutrition”) AND (“tailored Nutrition” OR “Medically tailored meals” OR “personalized senior Nutrition” OR “individualized senior nutrition” OR “Senior Nutrition intervention” OR “Senior Nutrition Program” OR (MH “Nutrition Education+”) OR (MH “Diet Therapy+”) OR “nutrition education” OR “diet counseling” OR “dietary education”)

An asterisk (*) indicates truncation, which was used to retrieve multiple word endings and variations (e.g., “United States*” retrieves United States, U.S., USA; “older adult*” retrieves older adult, older adults). Truncation was applied where supported by the database to enhance search sensitivity.

## Appendix B

**Table A2:** A summary of included studies on technology-delivered medically tailored meals (MTMs) and nutrition education for older adults in the United States.

Citation and Year	Study Design	Intervention	Technology Used	Primary Outcomes	Secondary Outcomes
Shah et al., (2023) [29]	Nonrandomized, two-arm intervention (remote vs. in-person)	Health literacy and cognitive training program delivered either remotely or in person over multiple sessions	Remote: video conferencing tools and digital training materials	Change in health literacy and cognitive performance from baseline to post-intervention and 8-week follow-up.	Feasibility metrics (enrollment, retention, completion rates), adherence to sessions, participant satisfaction
Chang AR et al., (2020) [45]	Mixed methods feasibility study	8-week remote dietary counseling with a Registered Dietitian Nutritionist (RDN) using either a study-specific app or a widely adopted commercial app as an adjunct	Smartphone app for dietary monitoring (study-specific or commercial), educational website, weekly telephone counseling (15–20 min), daily educational messages, weekly goal-reinforcement messages.	Change in sodium intake from baseline to 8 weeks	App utilization: App use was highest in the first 2 weeks and declined over the 8-week period. Counseling session participation: 85% of scheduled weekly RDN calls attended. Patient satisfaction: 90% reported being “very satisfied” or “extremely satisfied” with RDN counseling.
Dugas et al., (2018) [41]	Quasi-experimental, single-arm pretest–posttest pilot study	13-week diabetes self-management program using mobile technology + clinic visits	Samsung Galaxy Tab 3 with data plan, Fitbit One activity tracker, custom mHealth diabetes management app (Diasocial).	Change in HbA1c from baseline to 13 weeks	Physical activity levels, self-reported diabetes self-management behaviors, technology engagement, moderation effects of regulatory mod
Galiatsatos P et al., (2022) [48]	Pilot study with a pre–post intervention design	“Together in Care” program: Meals on Wheels partnership providing daily meal delivery, home safety inspection and modifications, medical supply provision, daily social engagement and scripted symptom surveys for 3 months post-hospitalization	Electronic tablet used by volunteers to record daily survey responses and relay in real-time to care manager; secure network for data transfer	Reduction in hospital expenditures	Reduction in readmissions (30 days, 3, 6, 12 months), COPD subgroup analysis, identification of social/health barrier
Go et al., (2022) [22]	Remote, decentralized, pragmatic randomized controlled trial	Up to 10 weeks of medically tailored meals (MTMs) post-discharge; subset randomized to receive additional virtual nutritional counseling (up to 3 sessions)	Virtual nutritional counseling via telehealth	All-cause hospitalization within 90 days after discharge	Hospitalization for heart failure, hospitalization for diabetes-related complications, all-cause mortality, all-cause ED visits, composite of utilization and death
Lee JJ, Sultana N, Nishita C, (2025) [42]	Quasi-experimental study with non-equivalent groups	Kūpuna U flexible congregate meal program with three models: Virtual (grab-and-go meals + online activities), Hybrid (grab-and-go meals + virtual and in-person activities), Traditional (in-person congregate meals + in-person activities)	Online platform for virtual activities and education	Change in food insecurity score	Changes in loneliness and self-rated health
Batsis JA et al., (2021) [16]	6-month pilot non-randomized controlled trial	26-week weight management program: 18 individual video nutrition sessions, 7 on-site group nutrition sessions, 75 min twice-weekly group exercise via video, aerobic activity prescription, Fitbit-based self-monitoring	HIPAA-compliant Zoom for video-conferencing; Samsung Galaxy A Tab with Amazon Firestick for TV mirroring; Fitbit Alta HR with Fitabase for data aggregation	Feasibility (recruitment, retention, attendance, Fitbit adherence), Acceptability (participant satisfaction	Changes in weight, BMI, waist circumference, physical function (30STS, 6 min walk, grip strength, gait speed), LLFDI score
LoBuono DL et al., (2021) [46]	Mixed-methods, convergent parallel design	Assessment of acceptance, perception, and digital competence for digital health to manage nutrition in PwPD and caregiver	Various digital health tools (videoconferencing, smartphones, internet apps, wearable devices, patient portals, dietary tracking apps)	Acceptance and perception of digital health for nutrition management	Digital competence scores, technology access/usage patterns
Yu L et al., (2022) [49]	Retrospective longitudinal cohort study	Food Access Pilot Project (FAPP): weekly home-delivered medically supportive meals or meal kits + optional monthly pantry box; telephone-based registered dietitian support; targeted to food-insecure PLHIV in 3 rural California counties	Online meal vendors (HelloFresh, Freshly, Amazon Pantry); phone counseling by RD	Change in food insecurity status; change in HIV viral suppression (VL < 200 copies/mL); change in CD4 ≥ 500 cells/mm^3^; change in continuous CD4 count	Change in proportion with undetectable viral load (VL < 40 copies/mL)
Krebs P et al., (2017) [37]	Pilot randomized controlled trial	Provider advice + brief counseling + tailored eHealth program on DVD based on ACS guidelines vs. advice and counseling alone	DVD-based interactive program with tailored feedback, survivor stories	Self-reported fruit and vegetable intake; self-reported physical activity	Intervention use, acceptability, satisfaction, qualitative feedback
Rodriguez MA et al., (2019) [38]	Randomized controlled trial	Tailored Behavioral Intervention (TBI): Monthly Trans theoretical model-based telephone counseling tailored to dietary stage of change; Non-tailored Intervention (NTI): Monthly general health education calls; Usual Care (UC): In-person visits only	Telephone counseling with computer-based counseling manual for TBI; telephone calls for NTI	Change in DASH diet adherence score from baseline to 6 months	Change in dietary Stage of Change (SOC) from baseline to 6 months
Salas-Groves E, 2024 [39]	16-week, single-center, parallel-group randomized controlled trial	Weekly web-based cooking demonstrations + biweekly nutrition education videos emphasizing protein intake from lean red meat vs. recipe handouts only	Web-based cooking videos; Nutrition education videos; Email delivery; vívofit 4 (Garmin) activity tracker	Change in muscle mass (g) via DXA	Protein intake (g/day), muscle strength (kg), daily steps, physical activity (min/week), cooking confidence, diet quality
Schrauben SJ et al., 2022 [44]	Single-center, single-arm study	8-week intensive dietary app–supported tele-counseling with dietitian (weekly calls), daily nutrition tips, weekly tailored feedback emails, educational website; maintenance counseling at 5 and 11 months, grocery store tour	MyFitnessPal app for dietary self-monitoring; study-developed educational website; telephone counseling	Change in mean 24 h urine sodium at 12 months vs. baseline	Changes in HEI-2015 score, BP, weight, urine potassium/phosphorus, albuminuria, protein intake, adherence
Sharma V, 2024 [47]	Mixed methods intervention study	Nutrition education and counseling program	Telephone-based counseling sessions	Improvement in diet quality scores	Changes in physical activity, weight, and BMI
Wood BS et al., 2024 [43]	6-month, non-randomized, non-blinded, single-arm pilot study (secondary retrospective analysis)	Multicomponent weight loss program with weekly 1:1 virtual dietitian session, monthly in-person group nutrition sessions, twice-weekly virtual 75 min physical therapist-led exercise classes, monthly in-person exercise sessions, plus independent aerobic and resistance training	Telemedicine via video-conferencing, Fitbit Alta HR for remote activity monitoring	Change in weight; change in 30 s sit-to-stand repetitions	6MWT distance, gait speed, LLFDI score, REAP-S diet quality score, caloric intake (ASA-24), fat mass %, skeletal muscle mass/weight, visceral adipose tissue
Su Y, Wu KC, Chien SY, Naik A, Zaslavsky O. 2023 [40]	Pilot randomized controlled trial	“Olitor” mobile app designed to enhance adherence to the Mediterranean diet for older adults with frailty, used ≥1× weekly for 3 months	Patient-facing mobile app (“Olitor”) with self-tracking, feedback, personalized recipes, in-app messaging; secure web-based admin dashboard	Adherence to Mediterranean diet score; insulin resistance	Retention, engagement, app quality ratings; intake of Mediterranean diet food groups; Mediterranean diet knowledge; physical performance (SPPB); behavior change measures (self-efficacy, self-regulation, social support, outcome expectations); anthropometrics; metabolic analytes (lipids, CRP)
